# Current Treatment Approaches and Global Consensus Guidelines for Brain Metastases in Melanoma

**DOI:** 10.3389/fonc.2022.885472

**Published:** 2022-05-05

**Authors:** Xiang-Lin Tan, Amy Le, Fred C. Lam, Emilie Scherrer, Robert G. Kerr, Anthony C. Lau, Jiali Han, Ruixuan Jiang, Scott J. Diede, Irene M. Shui

**Affiliations:** ^1^ Merck & Co., Inc., Rahway, NJ, United States; ^2^ Richard M. Fairbanks School of Public Health, Indiana University, Indianapolis, IN, United States; ^3^ Division of Neurosurgery, Huntington Hospital, Northwell Health, Huntington, NY, United States; ^4^ Seagen Inc., Bothell, WA, United States; ^5^ Integrative Precision Health, Limited Liability Company (LLC), Carmel, IN, United States

**Keywords:** melanoma, brain metastasis, immunotherapy, targeted therapy, treatment guidelines

## Abstract

**Background:**

Up to 60% of melanoma patients develop melanoma brain metastases (MBM), which traditionally have a poor diagnosis. Current treatment strategies include immunotherapies (IO), targeted therapies (TT), and stereotactic radiosurgery (SRS), but there is considerable heterogeneity across worldwide consensus guidelines.

**Objective:**

To summarize current treatments and compare worldwide guidelines for the treatment of MBM.

**Methods:**

Review of global consensus treatment guidelines for MBM patients.

**Results:**

Substantial evidence supported that concurrent IO or TT plus SRS improves progression-free survival (PFS) and overall survival (OS). Guidelines are inconsistent with regards to recommendations for surgical resection of MBM, since surgical resection of symptomatic lesions alleviates neurological symptoms but does not improve OS. Whole-brain radiation therapy is not recommended by all guidelines due to negative effects on neurocognition but can be offered in rare palliative scenarios.

**Conclusion:**

Worldwide consensus guidelines consistently recommend up-front combination IO or TT with or without SRS for the treatment of MBM.

## 1 Introduction

The global incidence of melanoma is increasing, accounting for 73% of skin cancer-related deaths (1, 2). Despite melanoma being the least common type of skin cancer, 60% of patients develop melanoma brain metastases (MBM), with a dismal median survival of 3 to 6 months (3, 4). Immunotherapy (IO), including anti-programmed cell death protein 1 (anti-PD1) and anti-cytotoxic T lymphocyte-associated protein 4 (anti-CTLA-4) therapies (5), and targeted therapy (TT) against BRAF V600 E/K mutations (BRAFi) and MEK/MAPK signaling pathways (MEKi) (6), have improved progression-free survival (PFS) and overall survival (OS) of patients with metastatic melanoma and reached median OS of up to 24.3 months (7). Delivery of precise doses of radiation using stereotactic radiosurgery (SRS) to discrete MBM and adjuvant doses of radiation to the post-surgical resection cavity have also significantly improved local intracranial disease control (8, 9).

In this review, we conducted a targeted literature review by focusing on the current modalities for the treatment of MBM as outlined in the consensus guidelines from the European Society for Medical Oncology (ESMO) (10), European Organization for Research and Treatment of Cancer (EORTC) (11), National Comprehensive Cancer Network (NCCN) (12), Cancer Council of Australia (CCA) (13), and Japanese Dermatological Association (JDA) (14). We further offered a comprehensive comparison of the consensus guidelines for each modality.

## 2 Methods

A targeted literature review for the treatment of MBM and the most recent international guidelines on the treatment of cutaneous melanoma with respect to MBM was performed. Guidelines reviewed included: 1) The National Comprehensive Cancer Network (NCCN) Clinical Practice Guidelines in Oncology, Cutaneous Melanoma, version 2.2021 (12); 2) The 2019 European Organization for Research and Treatment of Cancer (EORTC) recommendations on cutaneous melanoma diagnosis and treatment (11); 3) The European Society for Medical Oncology (ESMO) consensus conference guidelines on melanoma (10); 4) Evidence-based clinical practice guidelines for the management of MBM put forth by Cancer Council Australia (CCA) in 2020 (13); and 5) The 2019 melanoma guidelines of the Japanese Dermatological Association (14). The American Society of Clinical Oncology (ASCO) is currently preparing guidelines for the treatment of MBM but has not yet been published (15). References mentioned throughout this manuscript pertaining to the treatment of MBM were directly drawn from studies that were reviewed and referenced within the consensus guidelines themselves.

## 3 Results

### 3.1 Current Modalities for the Treatment of Melanoma Brain Metastases as Outlined in the Global Consensus Guidelines

#### 3.1.1 Role of Surgery

Surgery is recommended in the setting of large symptomatic lesions (> 3 cm diameter) presenting with mass effect, hemorrhage, or obstructive hydrocephalus. Patients with a single MBM, functional independence, limited or absent extracranial disease, should be offered surgery with palliative benefits (16, 17). MBM patients treated with immunotherapy and surgery achieve excellent local control rates (18). Similarly, patients with a single MBM treated with surgery plus whole brain radiotherapy (WBRT) have longer survival than WBRT alone (19, 20). Response to IO is associated with prolonged survival in patients who underwent resection of their MBM, while adjuvant WBRT does not (21).

#### 3.1.2 SRS and WBRT

SRS delivers a high dose of radiation to a focused target with high three-dimensional conformality and has proven efficacy at controlling a small number (< 4) of MBM lesions (with a total cerebral tumor volume of < 5 cubic centimeters) (8, 22–24). It has been suggested that multiple lesions, failure to treat with IO or TT, poorly controlled systemic disease, and intratumoral hemorrhage are predictors of poor response to SRS (23). A phase III randomized clinical trial (RCT) showed that adjuvant SRS boost to the surgical cavity significantly lowers local recurrence but does not improve OS (25).

WBRT was traditionally used to treat patients with multiple MBMs but only affords a small increase in median survival of 3.5 months, albeit before recent systemic therapy advances (26, 27). A pooled analyses of trials comparing WBRT to WBRT plus surgery showed no significant difference in OS (28) and patients treated with WBRT had decreased neurocognitive function (29). Furthermore, a multicenter RCT comparing WBRT plus surgery with surgery alone in 215 MBM patients did not demonstrate any clinical benefit for adjuvant WBRT and therefore adjuvant WBRT is no longer offered to patients (30, 31).

#### 3.1.3 Systemic Therapies Including IO and TT

Combination IO (anti-CTLA4 and anti-PD-1) and TT that inhibit BRAF V600 E/K and MEK (known to be mutated in approximately 40-50% of melanoma patients) are effective at treating MBM and prolonging PFS (5, 7, 32–34). The open-label, multicenter, single-arm phase II study CheckMate 204 suggested that combination IO nivolumab (nivo) plus ipilimumab (ipi) had clinically meaningful intracranial efficacy, concordant with extracranial activity in patients with at least one asymptomatic, measurable, non-irradiated BM (5). The anti-PD1 brain collaboration (ABC) trial also demonstrated clinically meaningful intracranial efficacy of combination IO nivo plus ipi (33). Similarly, the phase II multicentered COMBI-MB trial of combination TT dabrafenib plus trametinib in patients with BRAF V600 E/K mutant asymptomatic MBM demonstrated clinical safety with manageable symptoms (7). A recent systematic review and meta-analysis of combination IO, TT, and mono-agent IO in combination with radiotherapy for the treatment of MBM patients revealed that combination IO and TT had a similar intracranial response rate, while combination IO was associated with increased PFS and OS compared to mono-agent IO and combination TT (32).

#### 3.1.4 Combination of SRS Plus IO or TT

Multiple systematic reviews and meta-analyses have demonstrated a survival benefit of combining SRS with concurrent IO or TT compared to SRS alone (9, 35–40). As such, combination IO or TT are now recommended as upfront treatments followed by SRS and/or surgical resection of MBM. When combining SRS with TT, there should be a washout period of 3 to 5 days prior to commencement of SRS (41).

### 3.2 Review and Comparison of Worldwide Consensus Guidelines

In the second segment of this review, we summarize and compare the most recent global consensus guidelines published by ESMO, EORTC, NCCN, CCA, and JDA (**Table 1**). Of note, Canadian guidelines were omitted because they do not discuss the treatment of MBM. Comparison of guideline recommendations are subcategorized according to treatment modalities with the understanding that all current consensus guidelines state that most MBM patients will likely require multimodal combination therapies throughout their treatment course.

**Table 1:** Summary of published world guidelines for the treatment of melanoma brain metastases.

Treatment	NCCN Guidelines	ESMO Guidelines	EORTC Guidelines	CCA Guidelines	JDA Guidelines
**Immunotherapy/Targeted Therapy**	- Upfront IO for asymptomatic, low-burden intracranial disease. - TT in patients with BRAF V600 E/K mutations.	- IO for asymptomatic patients - TT for patients with BRAF V600 E/K mutations.	- IO preferentially offered. - TT in patients with BRAF V600 E/K mutations.	- First-line in asymptomatic patients with MBM. - Efficacy of IO/TT for symptomatic lesions is low.	- IO and TT are recommended, level C evidence.
**Neurosurgery**	- For *symptomatic* lesions in eloquent cortex, with hemorrhage, or in brainstem. - For patients who develop MBM while on systemic IO/TT. - Consider surgery in patients with symptomatic lesions after SRS that are not responsive to steroids.	- Surgical resection of solitary lesions given level C recommendation.	- SRS and surgery are considered equally effective at local control. - Surgical debulking procedures should be reviewed critically, as there is no evidence that they improve survival.	- Surgery reserved for patients with solitary, symptomatic lesion or with oligometastatic disease without extracranial metastases.	- Limited number of studies comparing IO/TT or SRS/surgery. - There is a need for RCTs in Japan to establish guidelines.
**Stereotactic Radiosurgery**	- SRS is the preferred radiationmodality.	- SRS is preferred for local control prior to systemic therapies for asymptomatic patients with 1-4 lesions < 4 cm diameter or 5-10 lesions < 3 cm in diameter. - Adjuvant SRS to surgical resection cavity should be considered to decrease local recurrence. - If considering concurrent SRS with IO/TT, early treatment is preferred over late SRS as salvage.	- Upfront SRS is recommended. - Surgery is an option when SRS is not possible.	- Upfront SRS recommended for asymptomatic patients with small number of asymptomatic lesions < 3 cm in diameter. - Adjuvant SRS to surgical cavity significantly improves local recurrence.	- No phase III RCTs have been completed to compare efficacy of IO/TT vs. SRS/WBRT vs. surgery.
**Whole Brain Radiotherapy**	- Adjuvant WBRT is not recommended after SRS/surgery. - Palliative WBRT is recommended only for palliative purposes when SRS is not feasible in patients with good KPS. - Hippocampal avoidance and memantine protocol should be considered to reduce neuro-cognitive toxicity.	- Not recommended due to lack of survival benefit and negative neurocognitive effects.	- WBRT should be abandoned as treatment option.	- May improve local control of SRS-treated lesions and distant lesions but has no survival benefit with negative neurocognitive effects. - Palliative WBRT may be used as last-line option in patients with multiple lesions who have failed SRS and systemic therapies.	- Lack of phase III RCTs necessary to comment on efficacy of WBRT.

IO, combination nivolumab + ipilimumab; **KPS**, Karnofsky Performance Score; **RCT**, randomized clinical trials; **SRS**, stereotactic radiosurgery; **TT**, combination dabrafenib + trametinib; **WBRT**, whole brain radiotherapy.

#### 3.2.1 Upfront and/or Subsequent Surgical Resection of MBM

Guidelines are inconsistent with regards to recommendations for surgical resection of MBM. The EORTC guidelines consider surgical resection as an option when SRS is not possible and that SRS is equally effective at achieving local brain control while being non-invasive, applicable to several lesions, repeatable, and provides early local control compared to surgical resection (11, 42). The NCCN (12) and CCA (13) guidelines state that patients with symptomatic lesions > 1 cm in diameter in non-eloquent cortex, resectable locations, should be offered surgical resection.

#### 3.2.2 Use of SRS

The NCCN currently recommends 15-24 Gy SRS in a single fraction to small tumors < 3 cm (43). SRS is typically not recommended for lesions > 4 cm, which may be treated with fractionated stereotactic radiotherapy (SRT), 24-27 Gy in 3 fractions or 25-35 Gy in 5 fractions (44, 45). Adjuvant SRS at 12-20 Gy may be applied to resection cavities < 5 cm (44) with fractionated SRT for larger cavities. TT should be held for ≥ 3 days before and after fractionated SRT and for ≥ 1 day before and after SRS to avoid toxicities associated with concurrent TT and SRS/SRT treatment (41, 46–50). EORTC considers SRS to asymptomatic MBM lesions < 3 cm (solitary or up to 5 lesions) to achieve superior early local control compared to surgical resection (42). ESMO recommends SRS for the treatment of limited asymptomatic MBMs (up to 4 lesions) with a maximum diameter of 4 cm or 5-10 lesions with the largest tumor < 10 mL in volume, < 3 cm in diameter, and a total cumulative volume of ≤ 15 mL (10, 51). The Australian guidelines recommend SRS in patients with a single or a small number of lesions (52–56). All guidelines except for the JDA recommend adjuvant SRS to the post-resection cavity based on two randomized trials evaluating effects of SRS to the resection cavity of multiple types of BM (25, 57). The JDA refrained from providing strong recommendations for adjuvant SRS to the resection cavity again due a lack of phase III randomized trials comparing SRS to local brain directed therapies (14).

#### 3.2.3 Use of WBRT

NCCN recommends considering palliative WBRT when SRS/SRT is not feasible in patients who have failed systemic therapy or in patients with signs and symptoms of leptomeningeal carcinomatosis. Hippocampal avoidance and memantine therapy should be considered to patients receiving WBRT to reduce neurocognitive toxicity (58). Adjuvant WBRT after resection or SRS/SRT is not recommended due to worsening cognitive decline following WBRT with no benefit in OS (57, 59). EORTC and EMSO guidelines recommend restricting WBRT to those few patients who have exhausted all systemic, SRS, and other local brain therapy options. All guidelines do not recommend treating patients with WBRT after surgical resection or SRS treatment for MBM.

#### 3.2.4 Use of IO and TT

The NCCN, ESMO, EORTC, and CCA recommend upfront combination IO (nivo + ipi) as the preferred initial treatment in patients with asymptomatic MBM < 3 cm, not requiring corticosteroids and who have not received prior systemic therapies. This recommendation is based on the study reporting high intracranial response rates using nivo + ipi in patients with previously untreated asymptomatic MBM (5). Anti-PD-1 monotherapy is not recommended, and systemic corticosteroids may negatively affect the efficacy of nivo + ipi and should be avoided in MBM patients (60). For patients with BRAF V600E mutations, combination BRAFi + MEKi should be considered. Brain-directed therapy is preferred in patients with symptomatic MBM as limited evidence exists supporting the effectiveness of upfront systemic therapies for symptomatic MBM (7, 60–62). In contrast, the JDA currently provides conditional recommendations for using IO or TT for the treatment of MBM patients due to the lack of phase III clinical trials comparing the efficacy of IO, TT, SRS, or surgery for the treatment of MBM, and that the existing phase II studies are limited by selection bias and small sample size (5, 33).

## 4 Discussion

The current iterations of consensus guidelines are limited to evidence gathered largely from relatively small, phase I and II clinical trials, retrospective case series, and observational studies (52–54, 63). CheckMate 204 was a phase II study evaluating the efficacy and safety of nivo + ipi in asymptomatic MBM patients with a relatively small sample size (n = 101 patients) and median follow-up of 14.0 months (5). Similarly, the phase II ABC study enrolled only 79 patients in 3 cohorts of patients treated with nivo or nivo+ipi, with considerable heterogeneity amongst the cohorts (33).

It is important to keep in mind when reviewing consensus practice guidelines that physicians in real-world practice may not always follow consensus guidelines. This may be due to a multitude of reasons, such as the availability of certain treatments or approval for their use by insurance providers. Studies using real-world evidence and observational data are being performed in an attempt to gain further understanding of actual treatment patterns (64). A recent study using the National Cancer Database (NCDB) of 3008 cases of MBM between 2011 to 2015 reported real-world outcomes of combination and the timing of IO with radiotherapy for MBM and showed longer survival in patients treated with combination IO with SRS/WBRT compared to SRS/WBRT alone and in patients receiving concurrent SRS and IO compared to non-concurrent therapy (40).

Limitations of this study included: The use of a retrospective database, precluding the ability to assess the benefit of IO given as second-line treatment since only IO given as first-line systemic therapy was recorded; And the exclusion of sociodemographic factors, disease factors, and treatment locations that could have limited a patient’s access to a specific treatment modality, which could have affected their outcomes (40). Thus, the ability to reference studies using real-world data could therefore serve as complimentary information to consensus guidelines for treating physicians.

Investigators are also now exploring novel combinations of multimodal therapies in MBM patients. These ongoing trials are mostly combining triplet therapy consisting of IO and TT with other novel small molecule inhibitors (65, 66). Current ongoing trials include: EMBRAIN-MEL (NCT03898908) combining Encorafenib plus Binimetinib before SRS; RadioCoBRIM (NCT03430947) combining vemurafenib plus cobimetinib after SRS; The phase III NIBIT-M2 study (NCT02460068) comparing the chemotherapy agent fotemustine alone versus combination fotemustine plus ipi alone or combination fotemustine plus ipi and nivo; And the phase II study combining vemurafenib and combimetinib with azetolizumab (NCT03625141). Ongoing trials are also evaluating the toxicity of SRS in combination with IO or TT, as previous studies have shown statistically significant differences in radiation necrosis and brain edema among patients receiving the combination, although data are inconsistent (34).

In summary, the evidence used to compile the current versions of the worldwide consensus guidelines show promise for improving the survival of patients with MBM who receive upfront concurrent combination IO or TT with SRS. The emergence of studies using real-world evidence could serve to further compliment consensus guidelines for the treatment of MBM.

## Author Contributions

X-LT, AL, FL, and JH contributed to conception and design of the review and wrote portions of the manuscript. All authors contributed to manuscript critique and revision and approved the submitted version.

## Conflict of Interest

X-LT, ES, RJ, SJD, and IMS are employees of Merck & Co., Inc. JH and AL are employed by Integrative Precision Health LLC. ES was also employed by Seagen Inc.

The remaining authors declare that the research was conducted in the absence of any commercial or financial relationships that could be construed as a potential conflict of interest.

## Publisher’s Note

All claims expressed in this article are solely those of the authors and do not necessarily represent those of their affiliated organizations, or those of the publisher, the editors and the reviewers. Any product that may be evaluated in this article, or claim that may be made by its manufacturer, is not guaranteed or endorsed by the publisher.
